# Toxicological Evaluation of Pooled Selected Fractions of *Mimosa invisa* and Protective Effect of Herbal Decoction

**DOI:** 10.4103/0971-6580.75873

**Published:** 2011

**Authors:** P.T.A. Usha, N. Gopakumar, A. M. Chandrasekhran Nair, N. Divakran Nair

**Affiliations:** 1 Department of Veterinary Pharmacology and Toxicology, College of Veterinary and Animal sciences, Mannuthy, Thrissur, Kerala-680651, India; 2 Centre of Excellence in Pathology, College of Veterinary and Animal sciences, Mannuthy, Thrissur, Kerala-680651, India

**Keywords:** *Boerhaavia diffusa*, *Hygrophila auriculata*, *Mimosa invisa*, *Tribulus terrestris*

## Abstract

The study was undertaken to evaluate the toxicity produced by the pooled selected fractions of *Mimosa invisa* in rabbits. An attempt was made to protect the animal from the toxicity using a decoction containing *Hygrophila auriculata, Tribulus terrestris* and *Boerhaavia diffusa*. Eighteen adult rabbits were divided into three groups of six animals each. Group I served as control. Group II received pooled fraction of *M. invisa* at 0.4 g/kg orally. Group III was administered with pooled fraction along with the decoction containing *H. auriculata, T. terrestris* and *B. diffusa* equivalent to 5 g/kg. The experiment was conducted for 20 days. Group II showed significant increase in biochemical parameters like alanine transaminase, aspartate transaminase, gamma glutamyl tranferase, creatine kinase, alkaline phosphatase, creatinine and urea, suggesting liver and kidney toxicity. Histopathological examination of the liver and kidney supported these findings. Heart also showed mild histopathological changes. Administration of decoction reversed the biochemical and histopathological changes, indicating the protective effect of decoction.

## INTRODUCTION

*Mimosa invisa* is a shrubby herbaceous plant, which is widespread in central and southern parts of Kerala. Toxicity due to consumption of this plant is very common in Kerala during rainy season. The earlier reports by Rajan *et al*,[1] indicated the toxic symptoms and pathological changes produced by this plant in calves. Detailed studies about the toxicity of this plant are lacking. Hence, an experiment was designed to study the toxicity produced by selected pooled fraction of *M. invisa*. An attempt has been made to protect the body from the toxicity with a decoction containing *Boerhaavia diffusa, Hygrophila auriculata* and *Tribulus terrestris*.

## MATERIALS AND METHODS

Alcoholic extract of *M. invisa* was prepared from the dried pulverized plant material. Alcoholic extract was again fractionated in chromatographic columns so that four fractions, namely, chloroform fraction, *n*-butanol fraction, aqueous fraction and water insoluble residue were separated. The pilot studies conducted revealed that the *n*-butanol fraction and aqueous fraction were toxic. Hence, these two fractions were pooled and the toxicity of this pooled toxic fraction was studied in this experiment. Eighteen adult rabbits procured from Small Animal Breeding Station, Kerala Agricultural University, were used for the study. The animals were divided into three groups of six animals each. Group I animals served as control, group II was administered with pooled toxic fraction (0.4 g/kg) of *M. invisa* and group III received pooled toxic fraction (0.4 g/kg) followed by a decoction containing *H. auriculata, T. terrestris* and *B. diffusa* (5 g/kg). Blood was collected before the administration of extract and at day 1, 3, 5, 10, 15 and 20. The serum was separated and biochemical parameters like alanine transaminase (ALT), aspartate transaminase (AST), gamma glutamyl tranferase (GGT), creatine kinase (CK), alkaline phosphatase (ALP), creatinine and urea were estimated. The analysis was conducted using Ecoline Kits manufactured by E. Merck Limited (India), in a semiautomatic blood analyzer (Microlab 200). The data were analyzed statistically by using “*t*” test.[2] All the animals were sacrificed at the end of the experiment (20^th^ day) and observed for gross pathological lesions in liver, kidney and heart. These tissues were preserved in 10% formalin for histopathological examination.

## RESULTS AND DISCUSSION

The results are presented in Table 1. Group II rabbits, which received the pooled toxic fraction of *M. invisa*, showed clinical symptoms like inappetence, dullness, lethargy and loss of body condition. These symptoms were observed from the second day onward. Similar symptoms were reported by Alex *et al*,[3] in heifers with *Mimosa* toxicity. Alikutty and Pillai[4] also reported reduced appetite in a clinical case of *Mimosa* poisoning in a buffalo. Group III (pooled toxic fraction + decoction) also showed similar symptoms from second day onward, but from the fifth day, they started taking feed and water and became very active gradually. This may be due to the effect of decoction, which reversed the changes produced by the toxin.

**Table 1:** Biochemical parameters before and after administration of pooled toxic fraction of *M. invisa* (0.4 g/kg) and decoction (5 g/kg)

Parameter	Group	Day						
		0	1	3	5	10	15	20
ALT	I	54.67±4.62	56±4.57	56±3.61	56.67±3.5	55.67±3.34	56.5±3.91	55.83±3.63
	II	55.5±5.69	85.5±8.05*	126.5±10.55**	136±13.06**	150.17±12.81**	162.67±8.09**	170.5±5.54**
	III	37±2.72	110±3.6	74.83±3.64*	65.67±4.51*	49.83±0.6	42±0.82	39±2.02
AST	I	50.17±3.11	49.5±2.58	49.83±3.0	50.5±3.21	50.83±2.44	51.17±2.65	50.5±2.74
	II	51±3.49	110±8.14**	154.17±15.08**	167.67±17.45**	188.5±11.65**	174.5±5.37**	186.83±5.78**
	III	45.17±1.20	81.67±2.68*	82±3.48*	66±2.10	57.83±2.37	42.17±1.33	42.83±1.91
GGT	I	5.5±0.56	5.67±0.42	5.5±0.43	5.5±0.43	5.17±0.31	5.17±0.48	5.33±0.56
	II	5.5±0.56	6.67±0.42	8.17±0.60**	9.83±0.87**	10.67±0.33**	11.5±0.22**	11.0±0.26**
	III	4.83±0.40	5.0±0.52	5.0±0.45	6.5±0.34	6.17±0.48	6.83±0.31	4.5±0.34
CK	I	132.67±7.0	134.5±6.39	129.67±6.63	135±6.64	132±7.89	133.5±5.6	131.5±7.0
	II	133.83±10.11	170.67±15.6*	199.33±13.76*	215.33±15.81**	170.33±6.98**	161.5±3.28**	140.5±3.43
	III	199.67±6.04	211±3.99	201.5±4.2	204±4.54	197.5±4.9	200.5±4.17	200.83±5.67
ALP	I	43.5±9.19	44.17±8.45	43.33±8.48	44±8.02	44±8.71	43.5±8.53	45.33±8.72
	II	42.83±10.64	46.5±11.05	62.17±12.25*	79.17±14.58*	63.67±10.63*	64.5±9.59*	53.67±8.19
	III	50.67±5.18	84±4.49	53.83±2.3	52.5±2.05	53.5±2.05	47.5±2.26	47.17±1.66
Creatinine	I	2.5±0.22	2.33±0.21	2.67±0.21	2.50±0.22	2.67±0.21	2.67±0.21	2.5±0.22
	II	2.17±0.31	4.67±0.33*	7.17±0.40**	8.33±0.49**	9.83±0.31**	10.80±0.40**	12.17±0.31**
	III	2.67±0.21	4.5±0.22*	5±0.26*	5.5±0.22*	4.83±0.17*	4.67±0.21*	2.5±0.22
Urea	I	59.83±2.87	58.83±2.41	59±2.41	58.33±2.73	58.67±2.93	58.33±2.90	60.00±2.92
	II	59.17±3.17	64.33±3.9	142.83±26.2**	157.33±23.92**	175.67±12.07**	195.83±8.99**	217.0±10.96**
	III	42.83±1.54	71.83±4.71*	69.17±2.12*	67.5±2.36*	63±1.61	55.35±3.48	47±5.01

* *P*<0.05;

** *P*<0.01;

*n* = 6; ALT, alanine transaminase; AST, aspartate transaminase; GGT, gamma glutamyl tranferase; CK, creatine kinase; ALP, alkaline phosphatase

There were significant changes in biochemical parameters in group II when compared with control animals (group I). A very significant (*P*<0.01) increase in serum ALT and AST was observed from the very next day after the administration of pooled toxic fraction of *M. invisa* in group II. Similar changes were observed with other plants. Flaoyen *et al*,[5] reported increased ALT levels after feeding flower stem of *Narthecium ossifragum* in goats. Flaoyen *et al*,[6] observed an increase in AST levels in sheep during experimental toxicity with *N. ossifragum*. According to Burtis and Ashwood,[7] in liver diseases associated with hepatic necrosis, the levels of ALT and AST rise even before the start of clinical symptoms. Hence, the increase in ALT and AST in the present study may be attributed to liver damage. In group III, a transient increase in AST and ALT levels followed by a gradual decrease was observed. Hewawasam *et al*,[8] observed a decrease in serum ALT and AST levels while studying the protective effect of *Hygrophila spinosa* extract in mouse liver injury induced by carbon tetrachloride and paracetamol. They suggested that this might be due to reduced leakage of enzymes from hepatocytes.

The GGT levels showed a significant increase in group II. Craig *et al*,[9] reported a continuous increase in GGT levels in toxicity due to pyrrolizzidine alkaloids. The group III animals exhibited considerable improvement in GGT levels when compared with group II animals.

The CK levels showed an increase followed by a decrease in Group II animals. Burtis and Ashwood (1996) explained that this may be due to muscular dystrophies. In group III animals, there was no significant increase in CK levels.

There was significant increase in ALP values throughout the experiment in group II animals, whereas group III animals showed a transient increase followed by normalcy. It may be due to hepatoprotective effect of *H. auriculata* as reported by Hewawasam *et al*,[8]

**Figure 1:**
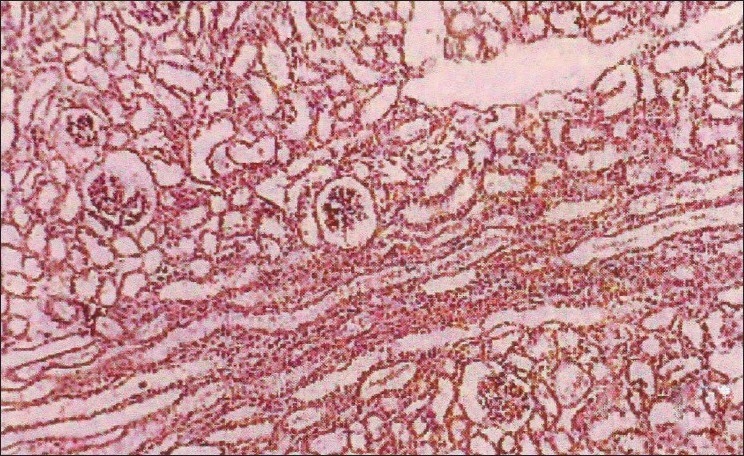
Kidney – pooled fraction – tubular dilatation, diffuse tubular degeneration, shrunken glomeruli (H and E, ×100)

**Figure 2:**
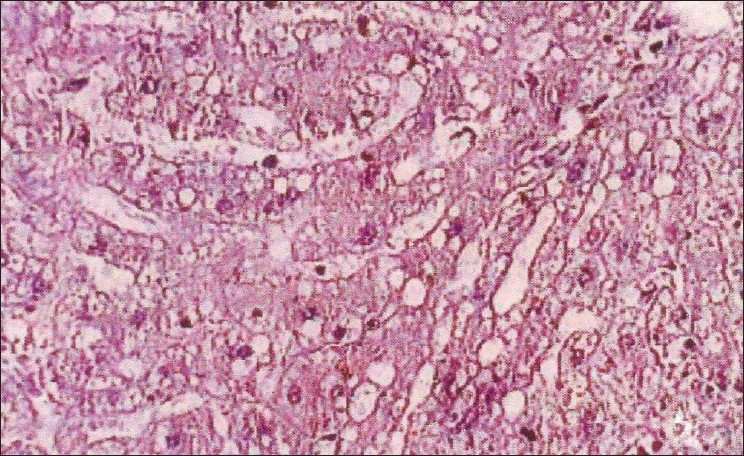
Liver – pooled fraction – fatty change, necrosis (H and E, ×400)

**Figure 3:**
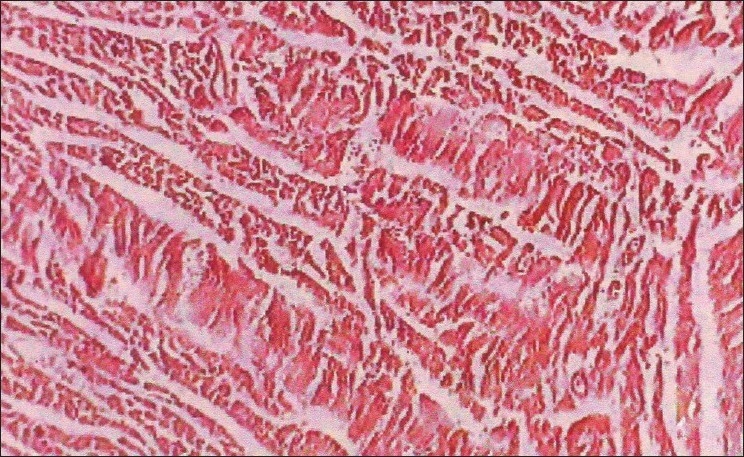
Heart – pooled fraction – focal myolysis, diffuse hyalinization (H and E, ×100)

**Figure 4:**
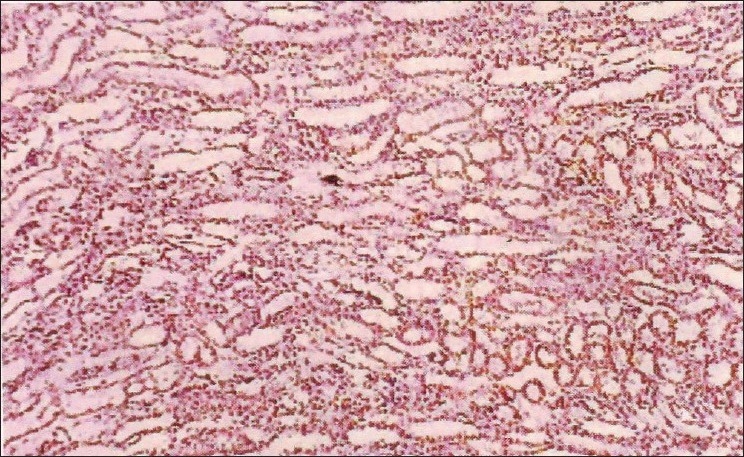
Kidney – pooled fraction + decoction – tubules with intact normal lining cells (H and E, ×100)

**Figure 5:**
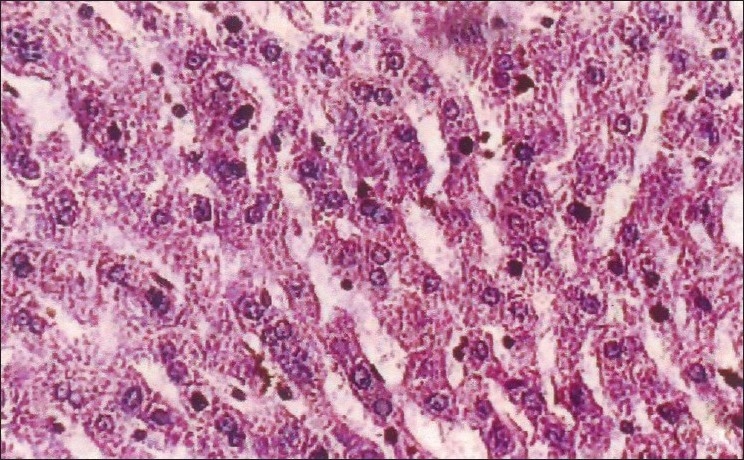
Liver – pooled fraction + decoction – sinusoidal dilatation, regenerative changes (H and E, ×400)

**Figure 6:**
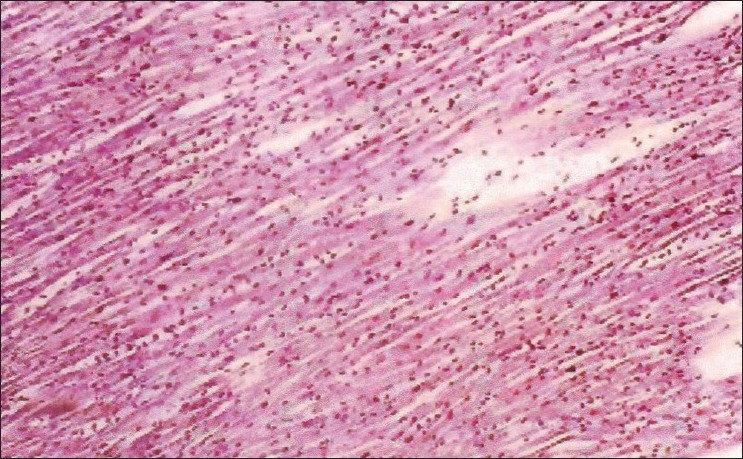
Heart – pooled fraction + decoction – intact heart muscle fibers (H and E, ×100)

The biochemical parameters, serum creatinine and urea showed a significant increase throughout the experiment in group II animals, and group III animals exhibited a decrease when compared to Group II animals. The rapid increase in urea and creatinine indicates typical impairment of kidney function.[10] The decrease in creatinine and urea observed in group III animals may be due to nephroprotective effect of *H. auriculata, T. terrestris* and *B. diffusa*. Gross pathological lesions include necrotic patches and petechial hemorrhages in the kidney and severe congestion and necrosis of the liver. Heart did not show any gross lesions. The histopathological examination of tissue collected from group II animals revealed tubular dilatation, diffuse tubular degeneration, shrunken glomeruli in kidney [Figure 1], fatty change and necrosis in liver [Figure 2] and focal myolysis and diffuse hyalinization in heart [Figure 3]. Postmortem examination of group III animals did not show any gross lesions; however, histopathological examination revealed regenerative changes [Figures 4–6].

## CONCLUSION

The results of the study reveal that the pooled fraction of *M. invisa* has the potential to produce nephrotoxic and hepatotoxic effects in rabbits. Administration of the decoction containing *H. auriculata, T. terrestris* and *B. diffusa* could protect the liver and kidney as evinced by the biochemical parameters and histopathological changes.
